# Decreased endemic malaria in Suriname: moving towards elimination

**DOI:** 10.1186/s12936-018-2204-x

**Published:** 2018-01-30

**Authors:** Edward D. van Eer, Gustavo Bretas, Hélène Hiwat

**Affiliations:** 1 Medical Mission, Zonnebloemstraat 45-47, Paramaribo, Suriname; 2 Independent Consultant, João Lira 84, 804, Leblon, Brazil; 3 Ministry of Health Malaria Programme, Anamoestraat 44bov, Paramaribo, Suriname

**Keywords:** Malaria elimination, Stable populations, *P. falciparum*, Accessibility of health services, Suriname

## Abstract

**Background:**

Suriname has moved from being the country with the highest annual parasite index in the Americas to one on the threshold of elimination. The progress toward elimination in the stable populations of Suriname between 2000 and 2015 is reviewed.

**Methods:**

Data was obtained from the Medical Mission and the Ministry of Health Malaria Programme case-reporting systems, and analysed with a focus on disease burden and differentiation of the disease geographically, by malaria species, age, gender, ethnicity, incidence and gametocytaemia.

**Results:**

Between 2000 and 2015 there were 57,811 locally acquired cases of malaria in the stable populations of Suriname. A significant reduction in indigenous malaria cases was observed from 2006 to 2015. The number of imported malaria cases saw a relative increase compared to the number of autochthonous cases. In 2015 over 95% of the cases reported in stable communities are imported, mainly from neighbouring French Guiana, a department of France. The overall decline in malaria case incidence followed the mass-distribution of free long-lasting insecticide-impregnated mosquito nets and increased awareness building efforts, improved access to malaria services as a result of the introduction of Rapid Diagnostic Tests and the implementation of active case detection in high risk areas. In addition, improved management of *Plasmodium falciparum* infections was achieved with the introduction of artemisinin combination therapy.

**Conclusions:**

The existence of a network of policlinics in the interior ran by Medical Mission, for the indigenous population, allowed the rapid implementation of the strategy in stable communities. The success of malaria control in Suriname indicates that the availability at local level, of prompt and adequate prevention, diagnosis and treatment is a key requirement for the elimination of malaria.

## Background

Malaria incidence in the world decreased with 41% between 2000 and 2015. About 212 million malaria cases and 429,000 deaths occurred in 2015 worldwide [1]. In the region of the Americas the number of confirmed malaria cases decreased from 1.2 million in 2000 to 390,000 in 2014 [2, 3]. In addition, malaria-related deaths decline by 79%. Three countries achieved zero indigenous cases between 2010 and 2015 and only two countries, -Haiti and Venezuela, saw an increase in their malaria burden [2].

**Fig. 1:**
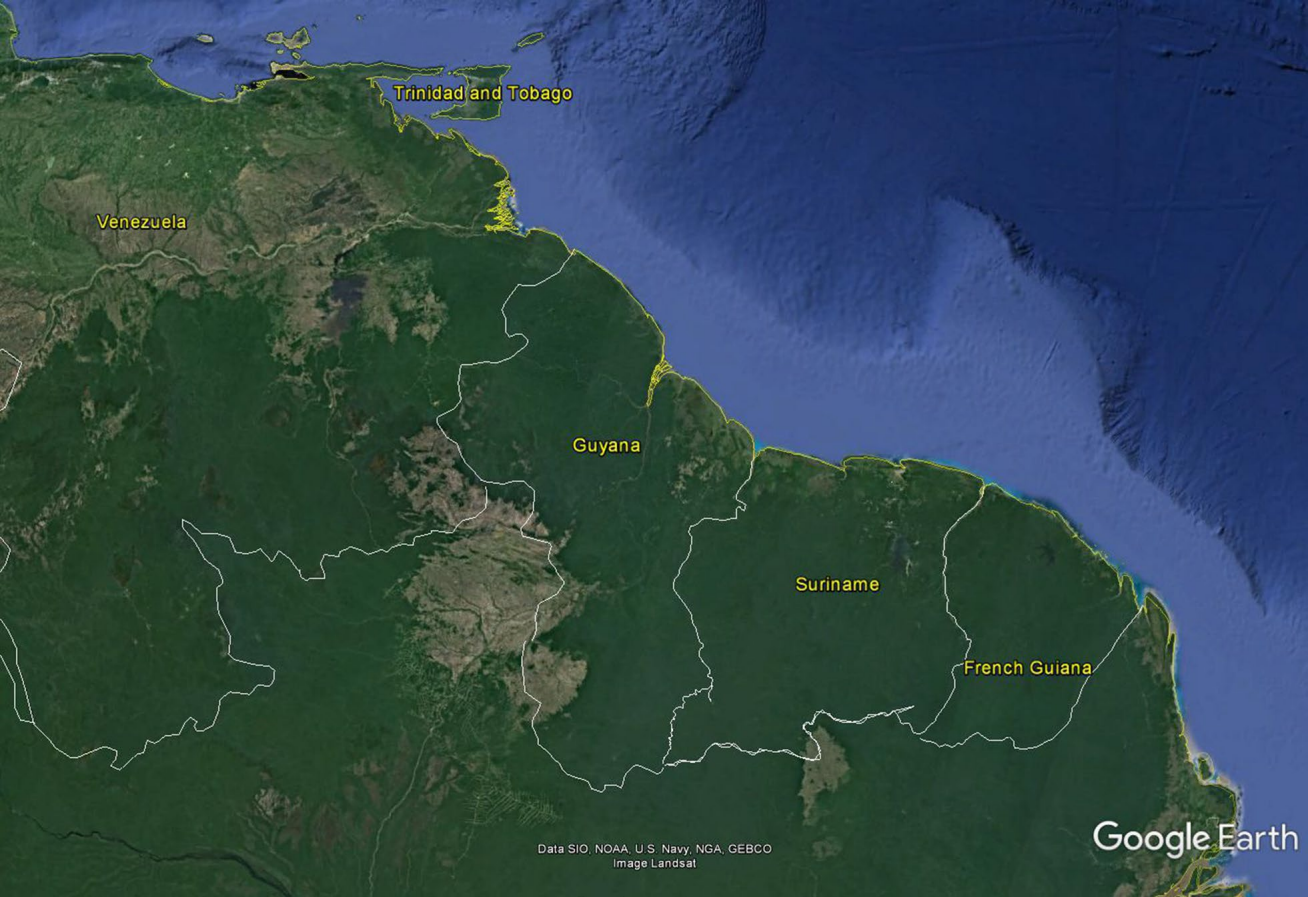
Map of Suriname related as part of the Guyana Shield countries on the Northeastern coast of South America (source: Google Earth, adjusted)

Suriname (Fig. 1) was responsible for the highest concentration of *Plasmodium falciparum* malaria in the Americas prior to 2006 [4]. The coastal area of Suriname has been free of malaria since 1968 [5] but malaria continued to be a problem in the interior of the country, especially in the Maroon and Amerindian village communities, located along the main rivers. Since 2006 Suriname experienced a significant decrease in malaria incidence, reaching near elimination levels by 2009 [6–8]. Transmission still occurs in the remote forested gold mining areas, with the mobile migrant miner population currently being most at risk [6]. The country is currently listed by the World Health Organization (WHO) among the countries with the potential to eliminate malaria by 2020 [9]. The most important interventions, implemented with support of the Global Fund to Fight AIDS, Tuberculosis and Malaria since 2006 were the introduction of artemisinin-based combination therapy (ACT) as first-line treatment for *P. falciparum* infections in 2004, free distribution of long-lasting insecticide-impregnated nets (LLINs) combined with an elaborate awareness campaign, a limited amount of active case detections (ACDs) in areas of high risk and during outbreaks, and indoor residual spraying (IRS) in “hot spot” areas in the stable populations. The IRS was discontinued rather quickly following the significant drop in national number of malaria cases in 2006 [7].

The study presented here describes the progress towards the malaria elimination phase in the stable populations in the interior of Suriname.

## Methods

### Area and population

Malaria has been a significant problem of the hinterland of Suriname since the 1970s. The predominant parasite species at that time was *P. falciparum* [7]. The southern hinterland, separated from the malaria-free northern coastal plains of the country by a so-called savannah belt, consisting of a white-sand formation, is covered with Amazonian rainforest, containing scattered villages, usually along rivers and creeks, with communities of Maroon and Amerindian inhabitants, the so-called stable population. The Maroons are of African descent and many of them have a Duffy negative phenotype which may prevents them for becoming infected by *Plasmodium vivax*. The population in the hinterland of Suriname consists of almost 50,000 people in the stable populations, making up to 10% of the country’s total population. In addition, there is a mobile population of variable size. The latter consists mostly of gold miners of foreign origin, mainly from Brazil. In the stable villages a specific health infrastructure exists for which the Medical Mission is responsible. The Medical Mission is a private religious based organization, with 50 health centres. The health centres deliver a package of Primary Health Care by means of health teams which consists of trained health-assistants and malaria microscopists supervised by medical doctors.

### Malaria diagnosis and surveillance system

The Medical Mission coordination centre in the capital city Paramaribo collects all malaria registration forms from the Medical Mission health centres in the interior. Malaria cases are coded and automated according to the species of malaria parasite (*P. vivax*, *P. falciparum* and *Plasmodium malariae*), the intensity of the infection, and presence of gametocytes. The malaria data are collected from a system of either malaria microscopists, who perform microscopic analysis of blood slides, or Medical Mission health care assistants, who obtain diagnosis by using rapid diagnostic tests (RDTs). All RDTs results are cross-checked by blood slide analysis.

Every person who present oneself at one of the health centres or outpatient clinics of the Medical Mission and who is suspected of malaria infection is subjected to a malaria test, both blood smear and a RDT. In addition, during suspected outbreaks of malaria in a settlement all local inhabitants are subjected to a malaria test as part of an Active Case Detection (ACD) survey. The blood smear is inspected by a malaria microscopist immediately on location or the following morning at the health centre. In the absence of a malaria microscopist in the health centres, management is done based on RDTs results and the corresponding blood smears are transported to the nearest health centre where a malaria-microscopist is present or to the Medical Mission coordination centre in the Paramaribo for cross-checking. The laboratory of the coordination centre is responsible for the quality control of testing that is performed on all positive malaria blood slides and a randomly selected 10% of the negative slides. The data collected for each suspected malaria case are: name of the person, age, gender, location where the disease symptoms arose and location where the patient stayed in the last 2 weeks, results of the thick smear and/or the rapid test, and if positive, the malaria parasite species and the intensity of the infection.

### Data analysis

A descriptive and observational study was conducted by analysing demographic and epidemiological data from the Medical Mission malaria cases registered by the Medical Mission health posts between 2000 and 2015. For this review the database of malaria cases of the Medical Mission was used. The malaria data in this digital database were derived from test results from all Medical Mission health centres and testing sites. The variables used for the analyses were date of diagnosis, region (MZ service region), country (country visit > 1 week before positive diagnose), *Plasmodium* species, number of trophozoites, presence gametocytes, sex, and age group. Only confirmed cases by thick smear were included in the analyses. The digital malaria data of the Medical Mission collected over the years 2000–2015 were analysed using Microsoft^®^ Excel 2013 and entered in Tableau software version 8.2 for tabulation and creation of graphs and distribution maps.

### Interventions

The chronology and scope of the implementation of major malaria prevention and control interventions by the Medical Mission is described and discussed in relation to the reported malaria incidence.

## Results

**Fig. 2:**
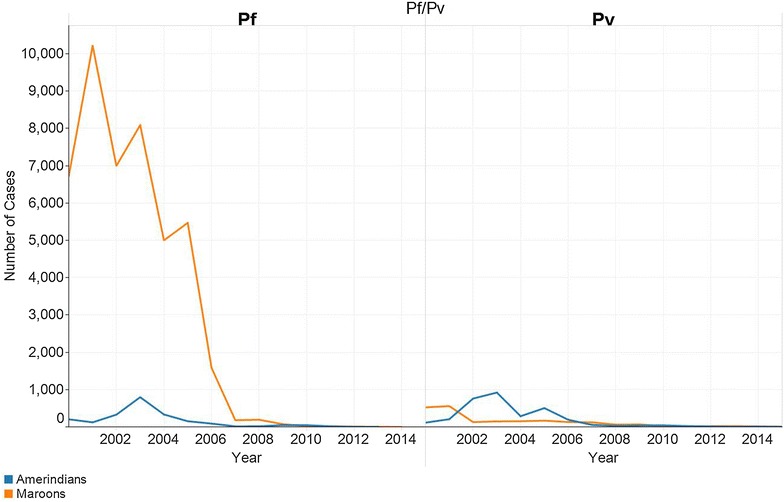
Number of *Plasmodium falciparum (Pf)* and *Plasmodium vivax (Pv)* cases between 2000 and 2015 by ethnicity in stable population in Suriname (source: data Medical Mission)

Both the Maroon and Amerindian communities experienced a reduction in malaria incidence over the study period. The decline of malaria incidence in the Maroon villages was much more pronounced than in the Amerindian villages. The proportion of *P. vivax* infections in Maroon malaria cases was very low. In the Amerindian population there were not many differences in the incidence of *P. falciparum* and *P. vivax* (Fig. 2). A significant reduction in indigenous malaria cases was observed from 2006 to 2015 (Fig. 2).

**Fig. 3:**
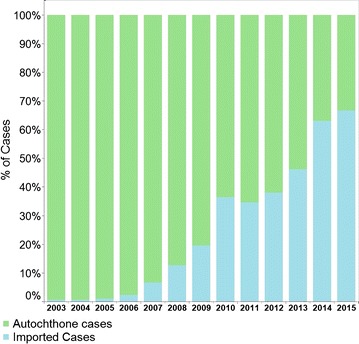
Number of malaria cases 2000–2015 by origin of infection of the stable population in Suriname (source: data Medical Mission)

The registration of the country, visited 1 week before the positive diagnose, started in 2003. In 2015 more than 95% of the cases originate from French Guiana (Fig. 3).

**Fig. 4:**
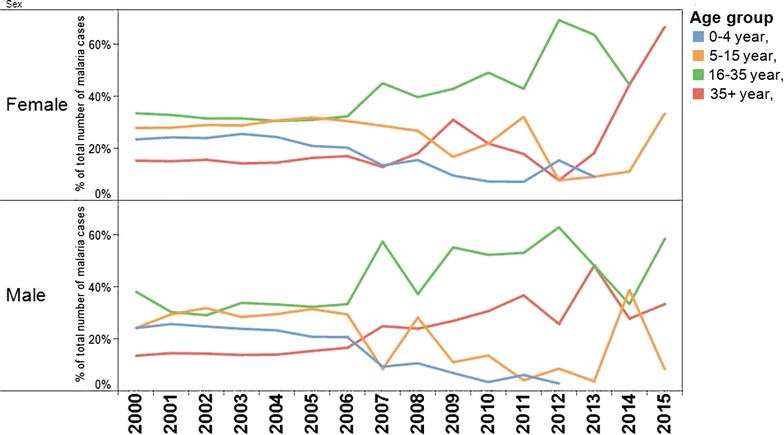
Percentage of malaria cases in the stable population in Suriname between 2000 and 2015 per age group and by sex (source: data Medical Mission)

The percentage of children infected with malaria declined in parallel with the decrease of malaria. In 2000 almost a quarter of the cases were in children under 4 years of age. After 2014 this was reduced to zero (Fig. 4). The proportion of men with malaria infection significantly increased, as the number of malaria cases decreased and the transmission in the villages approached zero (Fig. 4).

**Fig. 5:**
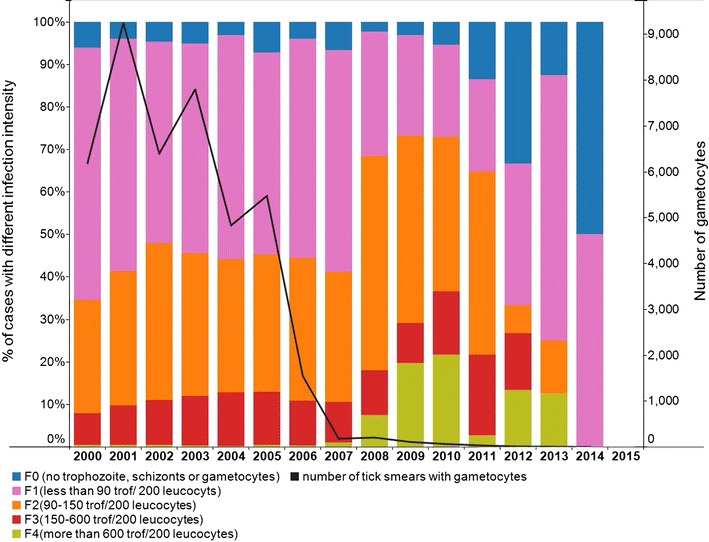
Intensity of *P. falciparum* infection- trophozoite/gametocyte in the stable population in Suriname between 2000 and 2015 (source: data Medical Mission)

With decreasing incidence of malaria and improved availability of diagnosis the intensity of infection in the case caused by *P falciparum*, based on number of trophozoites gradually increased (Fig. 5). From 2015 onward no *P. falciparum* infections were detected in the stable population.

A steep decrease of gametocyte count was observed from 2005 onwards (Fig. 5).

**Fig. 6:**
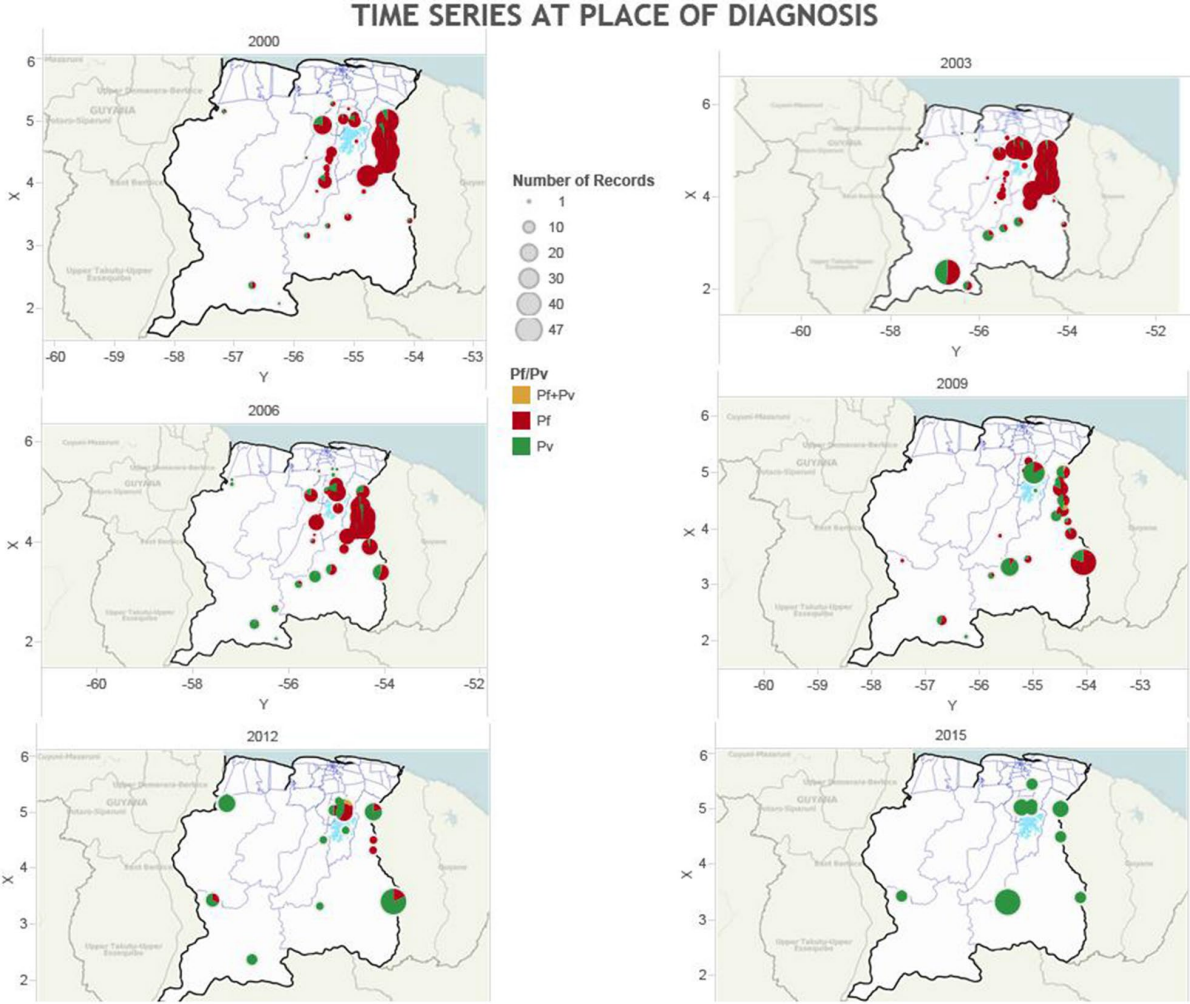
Time series of number of malaria cases in the stable population in Suriname between 2000 and 2015 per locality of diagnosis and by species (Pf = *P. falciparum*, Pv = *P. vivax*) (source: data Medical Mission)

Figure 6 shows the reduction in malaria incidence between 2000 and 2015 in the different regions in the hinterland.

### Interventions

**Fig. 7:**
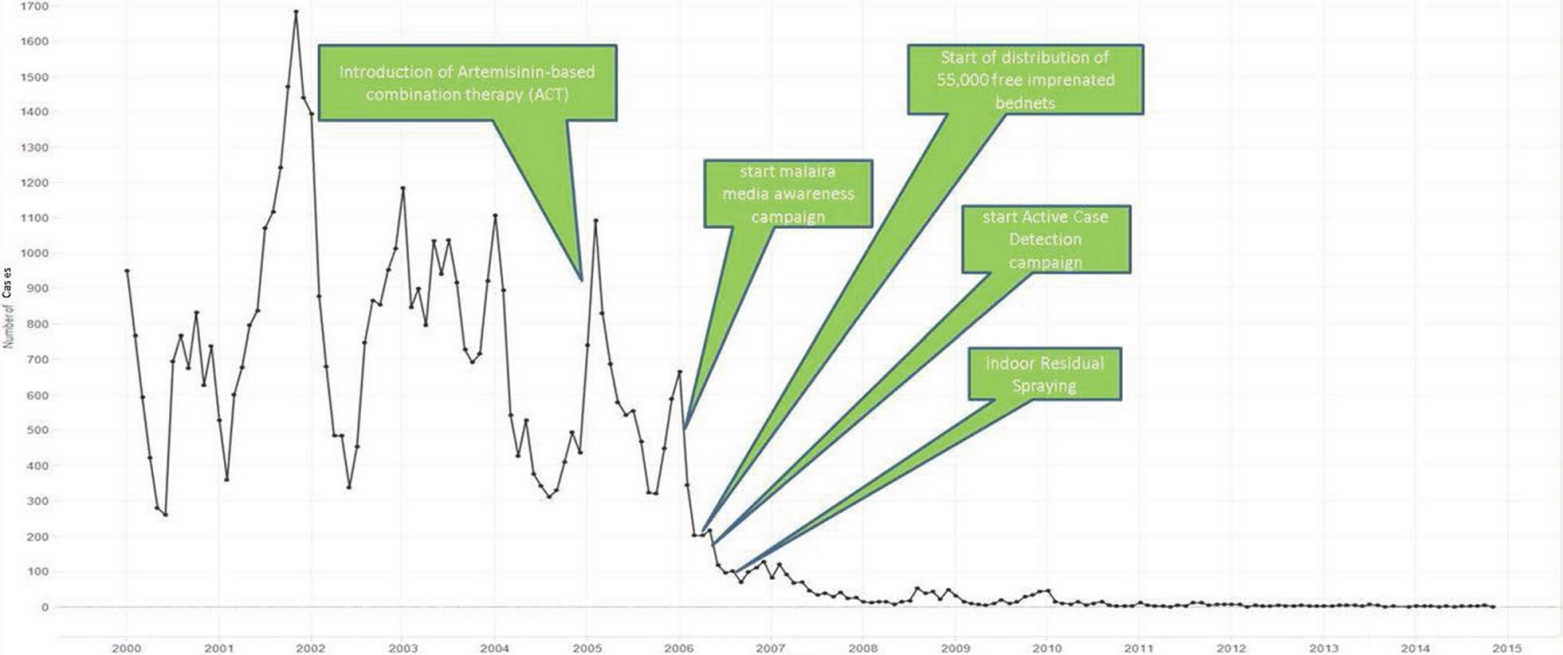
Malaria interventions in the stable populations in Suriname on a timeline of monthly malaria cases 2000–2015 (source: data Medical Mission)

The malaria prevention and control interventions are represented in a timeline in Fig. 7. In 2005 the artemisinin-based combination therapy was introduced nationally, replacing quinine as first-line treatment for *P. falciparum* infections. RDT’s were in use at the Medical Mission since 1999. In January 2006 malaria awareness campaigns were launched, which focused on prevention, especially with impregnated bed nets, mostly for children and pregnant women. In addition, it focused on the need for testing after experiencing symptoms. Since 2006, a protocol is in use establishing that if more than a certain number of positive malaria cases are recorded from a village an ACD campaign must be carried out to detect and treat other infected people. In 2006, over 55,000 LLINs were distributed among the stable population of the villages. The most significant reduction of malaria cases was established along the eastern border of the country with neighbouring French Guiana. This area had the highest malaria transmission risk prior to 2006. Most malaria cases were caused by *P. falciparum* as a result of the predominance of an Afro-descendent population with a Duffy negative phenotype.

## Discussion and conclusions

The stable populations in Suriname have seen a tremendous decrease in number of malaria cases over the 2000–2015 period. Malaria is at near-elimination level, but imported cases continue to emerge. The results show a strong decline of malaria in the period 2002–2007, which coincides with the upscale and adaptation of malaria prevention and control interventions. Characteristic is also the reduction of gametocytes after the introduction of ACT combination therapy. The largest decline in malaria incidence (Fig. 7) is likely to the introduction of ACT for *P. falciparum* infections. The decline was significant in the population of Afro-descendent, in which the majority of infections was due to *P. falciparum*. Several in vivo studies have suggested that ACT kill immature forms, but not mature gametocytes of *P. falciparum* [10]. Its use plus the prompt provision of diagnosis and treatment is thought to be the cause of the decline seen in the gametocyte count over the period (Fig. 5). With the previous treatment based on quinine, the frequency of finding gametocytes in the *P. falciparum* cases was high.

The decline in *P. vivax* infection (Fig. 2) with the national 14-day primaquine treatment scheme was achieved in stabile populations following adequate adherence. Despite the decrease in the total number of malaria cases it became clear that in the last 15 years men of working age are more likely to come into contact with other strata of malaria outside the stable villages. The localities of infection, especially in the recent years, are many times linked to gold mining situations in which interventions for malaria prevention and control are limited. In addition, imported malaria is becoming increasingly important. Migration by gold miners is a known problem in the region and in Suriname [6, 11, 12]. Knowledge of the location where the patient stayed in the last 2 weeks is to determine whether the case in an imported case or not. If the infection is acquired in an endemic country, but diagnosed in Suriname after the onset of the clinical symptoms it is qualified as an imported case. If the infection is acquired in the place where the symptoms arose it is an autochthonous case. Import in Suriname is coming for a large part from French Guiana [11, 12] and is suspected to be the result of lack of effective management of malaria migrants working in gold mining areas in the interior of this French overseas department [13]. The successes achieved along the border are thought to have had a spill-over effect in the neighbouring French territory [14, 15] with a parallel decrease of cases [11].

The increasing importance of miners as a risk group may explain that males of the age group 20 years and older are a growing portion of infected persons. Transmission in the villages of the stable population is clearly moving towards transmission outside of the villages, in this case the gold mining areas. Children from the stabile communities are generally not traveling to these risk areas, and are thus less likely to get infected. Percentage wise the number of parasites and thus the intensity of *P. falciparum* infection increased significant after implementation of the interventions, which may be due to a lack of pre-immunity. Immunity may be lost after 6–12 month without malaria [16]. One year after onset of the main intervention higher parasitaemia started to be visible. The effect was not accompanied by increase in mortality.

Malaria in the stable communities was eliminated without any strategy to deal with asymptomatic cases. Prevalence of asymptomatic cases is expected to have declined with declining malaria incidence, as has been the case elsewhere [17]. With decreased immunity of the population the likelihood of asymptomatic cases will have decreased.

The success of the Surinamese strategy for malaria control, indicates that it is possible to eliminate malaria with the proper combination of malaria services, consisting of good access to proper diagnosis and a quick response with good quality treatment. The most important prevention and control interventions were the introduction of ACT as first-line treatment for *P. falciparum* infections in 2004, free mass-distribution of LLINs to at-risk populations, and active case detection in areas of high risk and during outbreaks.

Spill-over of malaria from French Guiana as a result of miner movement, resulted in a higher number of imported malaria cases from French Guiana than the number of nationally acquired cases since 2014 (data Ministry of Health Malaria Programme Suriname). Suriname is adapting its strategies include distributing mosquito nets at the border, offering malaria diagnosis and treatment at border-crossing points and in remote mining areas in Suriname, and establishing cooperation and discussions with neighbouring counterparts to have impact on cross-border moving risk populations. Nevertheless, the WHO should play a strong role in motivating countries to take proper actions toward control and elimination in their territories, by effectively managing transmission in risk populations and areas.

In Latin America, malaria is strongly related to areas which have difficult access to diagnosis and treatment. This is especially true along the borders of Colombia and Ecuador, in the mining areas of Brazil and in countries with unstable political environments. Access to diagnosis and treatment is crucial and malaria control programmes in these areas should focus on providing quick diagnosis and proper treatment before working on other interventions. This is also one of the primary strategies of the WHO included in the Global Technical Strategy for Malaria 2016–2030 [18].

The near-elimination status in the stable populations in Suriname could be reached due to existence of the extensive network of health centres and test sites for the stable communities, maintained by the Medical Mission. Easy access to good quality diagnosis and adequate treatment, combined with quick response in outbreak situation has proven successful.

Although this study was carefully prepared, the authors are aware of its limitations such as human error when entering the data or missing data, because of the retrograde nature of the study, that no longer could be corrected or validated. Further there will be missed patients who travel to the capital or elsewhere for diagnosis and possible treatment. The lack of a scientific link of the interventions with the decrease of the malaria incidence may also be regarded as a limitation. A recommendation is that further research is needed to demonstrate this scientific link.
